# The Dual Role of Nod-Like Receptors in Mucosal Innate Immunity and Chronic Intestinal Inflammation

**DOI:** 10.3389/fimmu.2014.00317

**Published:** 2014-07-10

**Authors:** Daniele Corridoni, Kristen O. Arseneau, Maria Grazia Cifone, Fabio Cominelli

**Affiliations:** ^1^Department of Medicine, Case Western Reserve University, Cleveland, OH, USA; ^2^Digestive Health Research Center, Case Western Reserve University, Cleveland, OH, USA; ^3^Department of Life, Health and Environmental Sciences, University of L’Aquila, L’Aquila, Italy

**Keywords:** NOD-like receptors, inflammasome activation, inflammation, innate immunity, IBD

## Abstract

Nucleotide-binding and oligomerization domain NOD-like receptors (NLRs) are highly conserved cytosolic pattern recognition receptors that play, in combination with toll-like receptors, a critical role in innate immunity and inflammation. These proteins are characterized by a central oligomerization domain termed nucleotide-binding domain, and a protein interaction domain containing leucine-rich repeats. Some NLRs, including NOD1 and NOD2, sense the cytosolic presence of conserved bacterial molecular signatures and drive the activation of mitogen-activated protein kinase and the transcription factor NF-κB. A different set of NLRs induces caspase-1 activation through the assembly of large protein complexes known as inflammasomes. Activation of NLR proteins results in secretion of pro-inflammatory cytokines and subsequent inflammatory responses. The critical role of NLRs in innate immunity is underscored by the fact that polymorphisms within their genes are implicated in the development of several immune-mediated diseases, including inflammatory bowel disease. Over the past few years, the role of NLRs in intestinal homeostasis has been highlighted, however the mechanism by which dysfunction in these proteins leads to aberrant inflammation is still the focus of much investigation. The purpose of this review is to systematically evaluate the function of NLRs in mucosal innate immunity and understand how genetic or functional alterations in these components can lead to the disruption of intestinal homeostasis, and the subsequent development of chronic inflammation.

## Introduction

The innate immune system plays a pivotal role in the early induction of host defense mechanisms following exposure to pathogens. Innate immunity can broadly be classified into sensor, effector, and regulatory functions. Sensor activation of innate immune cells provides a primary host response and triggers the downstream effector and regulatory functions. This innate immune response is achieved through the recognition of microbial agents by pattern recognition receptors (PRRs), which include toll-like receptors (TLRs) and NOD-like receptors (NLRs). PRRs detect and translate signals of evolutionarily conserved pathogen-associated molecular patterns (PAMPs) into rapid host defenses, triggering sequential activation of intracellular signaling pathways that lead to induction of a range of cytokines that prime the adaptive immune response for long lasting-protection (1, 2). Moreover, it is now evident that PRRs also sense damage-associated molecular patterns (DAMPs), which are non-microbial ligands generated primarily from stress signals, suggesting that PRRs are important for both microbial defense and, when their signaling become dysfunctional, the pathogenesis of non-infectious inflammatory diseases (3, 4).

**Figure 1:**
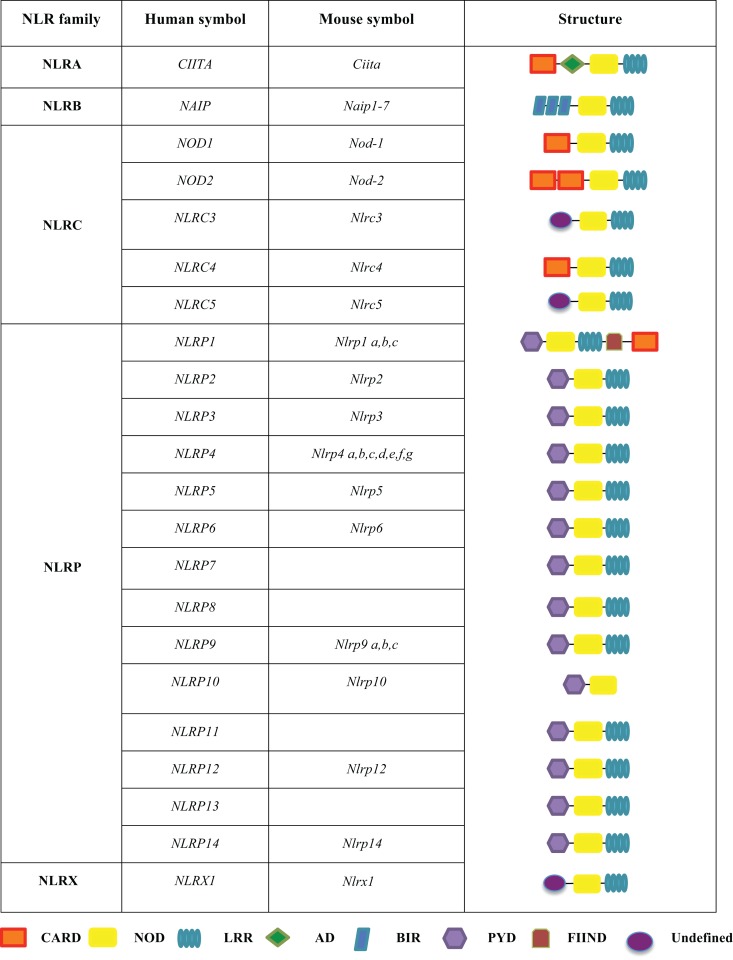
**The human and mouse NLRs family**. The NLR family is subdivided into five sub-families, including NLRA, NLRB, NLRC, NLRP, and NLRX. These proteins share common domains, such as a CARD caspase recruitment domain, a NOD nucleotide-binding oligomerization domain, a LRR leucine-rich repeat, an AD acidic transactivation domain, a BIR baculoviral inhibition of apoptosis protein repeat domain, a PYD pyrin domain, and a FIIND domain with function to find.

The NLRs are a group of conserved intracellular PRRs that play a pivotal role in innate immunity. The NLR family includes 22 identified protein members in humans and approximately 33 NLRs genes in mice (5) (Figure 1). The structural features of NLRs are characterized by a central nucleotide-binding oligomerization (NOD) domain, which mediates the self-oligomerization occurring during activation (6), a variable N-terminal protein–protein interaction domain, defined by the caspase recruitment domain (CARD), and a C-terminal leucine-rich repeat (LRR) that detects PAMPs. Based on the variation in their N-terminal domain, the NLRs family can be further subdivided into five families: NLRA or Class II transactivators (CIITA); NLRB or neuronal apoptosis inhibitor proteins (NAIPs); NLRC, which includes the CARD-containing molecules (NOD1, NOD2, and NLRC3-5) and the NLRP proteins (NLRP 1–14); NLRX, an additional sub-family that has no homology to the N-terminal domain of any of the other four subsets and consists of one member; and NLRX1, which is located within the mitochondria (5, 7–10) (Figure 1).

The physiological importance of NLRs in maintaining a finely balanced immune response becomes apparent when signaling derived from these components is dysregulated due to functional or genetic defects. An example of such dysregulation is during the development of several chronic inflammatory diseases, such as inflammatory bowel disease (IBD) (11–13).

## Innate Immune Response in IBD

Idiopathic IBD includes two major forms of chronic intestinal disorders: Crohn’s disease (CD) and ulcerative colitis (UC). Genetic, environmental, and/or epithelial barrier dysfunctions are all involved in the pathogenesis of IBD, and represent the sustained activation of mucosal immune responses. The pathogenesis of IBD was originally attributed to an overly aggressive adaptive immune response against luminal antigens. However, a paradigm shift within the past 10 years has led to the novel hypothesis that this chronic, relapsing inflammatory disease of the gut more likely results from a primary defect in intestinal innate immunity (14–16). In this context, several studies have provided very important insights into the role of innate cytokine-driven pathways during chronic intestinal inflammation. Interestingly, several innate cytokine may play a dichotomous role such that their dysregulation (overproduction or underproduction) could cause immunologic dysfunction and chronic intestinal inflammation (17–19). This recurrent concept is seen with innate cytokines (TNF-α, IL-1β, and IL-18) and signaling molecules [NF-κB and mitogen-activated protein kinase (MAPK)] that have long been associated with pro-inflammatory immune states (20). Activation of these pathways is mostly induced upon PAMP sensing by PRRs, including NLRs, to generate acute inflammatory responses that eliminate excessive numbers of bacteria and maintain mucosal homeostasis. Thus, underproduction of innate cytokines and signaling molecules during early, acute phases of disease could result in ineffective bacterial clearance and chronic intestinal inflammation. Alternatively, inappropriate activation of PRRs during later, chronic phases of intestinal inflammation could result in continuous stimulation of signaling pathways and overproduction of pro-inflammatory cytokines, with a similar end result of continued chronic intestinal inflammation.

The most compelling support for the concept that a primary defect in innate immunity leads to IBD comes from the clear genetic association between CD and carriage of polymorphisms within the *NOD2* gene (a component of the NLRC family of proteins), which represents the most frequent genetic defect in CD (13, 21–23). Consistently, dysregulation of other members of the NLR families have been associated with increased susceptibility to intestinal inflammation in both humans and animal models. Therefore, while NLRs are widely expressed in several tissues in both human and mice, in this review we provide an overview for those that have been shown to be expressed at the intestinal level and have a role in the pathogenesis of intestinal inflammation.

## Non-Inflammasome Forming NLRs and Intestinal Inflammation

### NOD2

**Figure 2:**
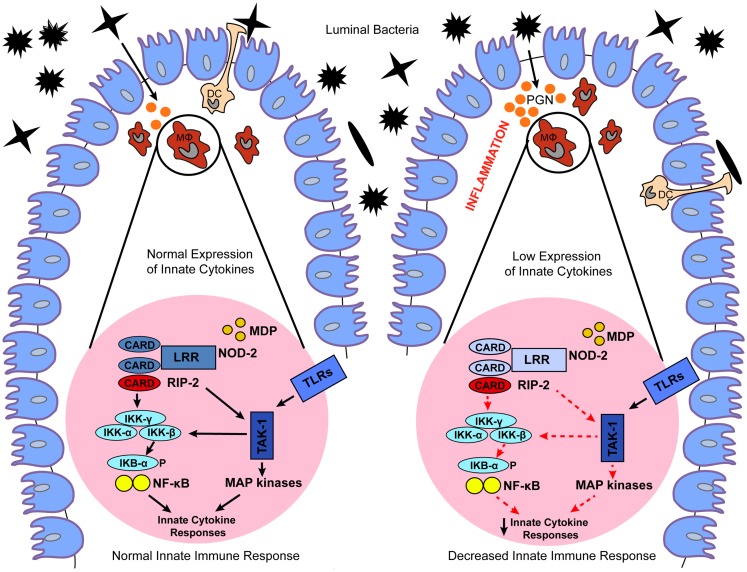
**Proposed models for the role of dysregulated NOD2 signaling in IBD**. Under normal conditions, NOD2 expressed in macrophages (MΦ) and dendritic cells (DC) recognizes MDP, a breakdown product of peptidoglycan (PGN) present in the cell wall of Gram-positive and Gram-negative bacteria. Exposure to MDP causes a conformational change in NOD2 that promotes binding of the dual specificity kinase RIP2 through homotypic CARD–CARD interactions. Binding to NOD2 promotes RIP2 kinase activation, and the NOD2–RIP2 complex then initiates a large signaling platform. Specifically, RIP2 activation promotes activation of a TAK1 complex that culminates in the activation of IκB kinase (IKK) complex, and IκBα phosphorylation and degradation. The NOD2:RIP2 complex also leads to activation of MAP kinases. This results in expression of innate cytokines, which promote normal bacterial clearance and maintain intestinal homeostasis. In contrast, genetic or functional defects in NOD2 cause a loss-of-function on downstream NOD2 signaling, which ultimately leads to decreased expression of innate cytokines. Decreased NOD2 function may result in a failure to respond to bacteria, facilitating their invasion and interaction with the gut mucosal immune system, which culminates in chronic intestinal inflammation typical of IBD.

NOD2 is a member of the NLRC proteins family that is expressed in both the hematopoietic and non-hematopoietic cellular compartments (24). The expression of NOD2 is more localized to the hematopoietic compartment, mostly in antigen presenting cells (APCs), however, NOD2 expression can also be upregulated in epithelial cells, including those of the gastrointestinal tract, upon the induction of pro-inflammatory stimuli, such as TNF-α and IFN-γ (25). NOD2 recognizes muramyl-dipeptide (MDP) a breakdown product of peptidoglycan present in the cell wall of Gram-positive and Gram-negative bacteria (5) (Table 1). Exposure to MDP causes a conformational change in NOD2 that promotes its oligomerization through the central NACHT domain and binding of the dual specificity kinase RIP2 through homotypic CARD–CARD interactions (26). Binding to NOD2 promotes RIP2 kinase activation, and the NOD2–RIP2 complex then initiates a large signaling platform. Specifically, RIP2 activation promotes a TAK1–TAB2–TAB3 complex that culminates in the activation of the IκB kinase (IKK) complex and IκBα phosphorylation and degradation. The NOD2:RIP2 complex also activates the MAPKs such as ERK-1, ERK-2, JNK, and p38 (27–29) (Figure 2; Table 1). This results in expression of pro-inflammatory cytokines, chemokines, microbial factors, and induction of adaptive immunity and regulatory T-helper 2 (Th2) type immune responses (29). In addition, as recently demonstrated, activation of NOD2 influences major histocompatibility complex (MHC)-cross presentation, autophagy induction, and resistance to intracellular bacterial infection (30–32). Thus, while most well-known for its acute signaling effects, NOD2 activation causes a variety of cellular changes *in vivo* that are also important for immunologic homeostasis.

**Table 1:** **NLR family members studied in IBD**.

NLR	Agonist	Pathway	Studied in IBD (Reference)
NOD1	iE-DAP		(41–43, 46)
	d-Lactyl-l Ala-γ-Glu-*meso*-DAP-Gly	NF-κB	
	Heptanolyl-γ-Glu-*meso*-DAP-Ala	MAPK	
	GM-tripeptide		
NOD2	MDP	NF-κB	(13, 16–19, 21, 23, 33, 34)
	MurNAc-I-Ala-g-d-Glu-I-Lys	MAPK	
NLRP3	LPS	Caspase-1	(71–74)
	MDP	
	LTA	
	Bacterial RNA	
	Viral RNA and DNA	
	Uric acid crystals	
	Silica	
	Asbestos	
NLRP6	Unknown	Caspase-1	(81, 83, 84)
NLRP12	Unknown	Caspase-1	(75, 89)
NLRC4	Flagellin from *Salmonella, Legionella, Listeria, Pseudomonas*	Caspase-1	(84, 104, 105)
	Type III and type IV secretion systems	
CIITA	Unknown	Transcriptional regulation of MHC	(111–113)

Detailed mapping of chromosome 16 identified polymorphisms within the *NOD2* gene (also designated *CARD15*) as the most frequent genetic alterations associated with CD. The CD-associated NOD2 polymorphisms are represented by a frameshift mutation in the LRR domain region (L110fsinsC), leading to partial LRR truncation, as well as the SNPs R702W and G908R, and the S431L and NN852S polymorphisms (16, 21, 33, 34). The associated risk is dose-dependent, with heterozygous carriers of these *NOD2* gene polymorphisms harboring a 2- to 4-fold increased risk of CD, and homozygous or compound heterozygous carriers having a 20- to 40-fold increased risk. Notably, the CD-associated *NOD2* gene polymorphisms cause a loss-of-function in the NOD2 pathway. To date, the exact mechanism explaining the effects of the loss-of function polymorphisms on downstream NOD2 signaling and CD have yet to be fully elucidated. It has been postulated that decreased NOD2 function manifests itself in a failure to respond to pathogens, facilitating invasion of bacteria and abnormal interaction between the gut mucosal immune system and luminal antigens, which culminate in chronic intestinal inflammation (13) (Figure 2).

An important point to note is that CD patients who do not possess genetic mutations in *NOD2* (nearly 85% of cases) may still have a separate functional defect somewhere along the NOD2 signaling pathway that produces the same downstream effect of NOD2 signaling dysfunction. In support of this concept, our group has shown that the SAMP1/YitFc (SAMP) mouse model of spontaneous CD-like ileitis fails to respond to MDP administration by displaying decreased innate cytokine production and dysregulated NOD2 signaling before the onset of disease, despite their wild-type *NOD2* genotype (13, 23). In line with these results, a recent study in humans has identified a subset of CD patients who do not carry NOD2 mutations but fail to produce innate cytokine in response to MDP, similar to SAMP mice (Nunez et al., personal communication).

During the different stages of chronic intestinal inflammation, it appears that NOD2 may play a dichotomous role such that any deviation, either positively or negatively, could cause an immune dysfunction that leads to inflammation (17–19). In fact the NOD2 signaling pathway appears to be a critical regulator of NF-κB-induced pro-inflammatory proteins, as the expression of both NOD2 and pro-inflammatory cytokines increases considerably upon inflammatory stimulation (35). It has been shown, for example, that pediatric CD patients who are wild-type for the *NOD2* genotype have overexpression and hyperactivity of NOD2 and its obligate kinase RIP2 in their small intestines (36). In addition, IL-10-deficient mice, which normally develop colitis, are protected from developing severe chronic colitis when a deletion of the NOD2 gene is also introduced into these mice. Moreover, macrophages isolated from IL-10 deficient mice display enhanced NOD2-dependent pro-inflammatory activity in which the well-known synergistic activity of MDP and TLR ligands is intrinsically hyper-responsive to bacterial stimulus, contributing to the development of colitis (37). In this context, it has been postulated that inhibition of the NOD2:RIP2 complex signaling in the presence of a wild-type NOD2 genome may be efficacious (17). These findings allude to complex and opposing roles for NOD2 in the pathogenesis of chronic intestinal inflammation, strictly dependent on the phase of disease. Therefore, it appears that NOD2 plays a dual role in the pathogenesis of chronic intestinal inflammation, by protecting the intestinal mucosa under normal physiological conditions and inducing the production of pro-inflammatory cytokine during the chronic phase of gut inflammation.

### NOD1

NOD1 is expressed in a wide variety of cells in both the hematopoietic and non-hematopoietic compartment. Within the non-hematopoietic compartment, NOD1 is highly expressed in intestinal epithelial cells where it plays an important role in maintaining gut homeostasis by regulating host primary responses to normal gut microbiota and pathogen (38). NOD1 shares the same structure of NOD2 with exception of the amino-terminal domain, which contains a single CARD, compared to the tandem CARDs contained within the NOD2 domain (Figure 1). The NOD1 LRR region has been implicated in the recognition of the diaminopimelate (DAP)-containing GlcNAc-tripeptide muropeptide found mostly in Gram-negative bacterial peptidoglycans (39) (Table 1). As observed for NOD2, exposure to NOD1 ligand causes a conformational change that promotes its oligomerization through the central NACHT domain and binding of the dual specificity kinase RIP2 through homotypic CARD–CARD interactions. This results in activation of NF-κB and MAPK pathways and regulation of acute inflammatory responses (40) (Table 1).

NOD1 gene has been mapped to chromosome bands 7p14–p15, a region which was previously reported to contain an IBD susceptibility locus. This focused much attention on the *NOD1* gene as a candidate factor associated with increased susceptibility to IBD. An insertion/deletion polymorphism (rs6958571) was identified within the *NOD1* gene, and a number of studies were conducted to investigate the association of this polymorphism with human IBD (41). However, these studies had conflicting results (42). To date, the detailed functions of this polymorphism remain unclear, and further studies on the function of *NOD1* insertion/deletion polymorphism are required.

Although an insertion/deletion functional polymorphism has not definitively been associated with IBD, animal studies have demonstrated an important role for NOD1 in experimental intestinal inflammation. For example, Cheng et al. demonstrated an increase in dextran sodium sulfate (DSS)-induced epithelial injury, intestinal permeability, and inflammation in the absence of NOD1 signaling (43). The increased intestinal inflammation was associated with increased epithelial proliferation within colonic crypts, suggesting that NOD1 may be important in maintaining the integrity of the intestinal epithelium to protect against injury and inflammation. One possible explanation is that NOD1 stimulation, as mentioned above, results in the activation of NF-κB, a positive regulator of cell survival (43, 44). In fact, NF-κB plays a crucial role in maintaining the integrity of the intestinal epithelium, specifically through the adaptor molecule NEMO, such that its deletion in mice results in increased intestinal epithelial apoptosis and spontaneous colitis (45). Moreover, NOD1-deficient mice possess an elevated risk of developing colonic cancer after experimental treatment with azoxymethane (AOM)–DSS. This increased susceptibility to develop tumors in NOD1-deficient mice is strictly dependent on signals deriving from the microbiota. Accordingly, antibiotic eradication of host microbiota in NOD1-deficient mice before the experimental induction of colitis resulted in reduced frequency of developing polyps (43). Finally, a study demonstrated that NOD1 recognizes *Clostridium difficile* and regulates intestinal neutrophil recruitment, a typical local inflammatory response to infection that has been demonstrated to be defective in NOD1-deficient mice (46). This suggests that loss of NOD1 mediates a loss of specific local inflammatory responses resulting in more severe and uncontrolled intestinal inflammation in response to pathogens.

## Inflammasome Forming NLRs and Intestinal Inflammation

### NLRP3

NLRP3, a member of the NLRP protein family, is expressed in response to inflammatory stimuli mainly within the hematopoietic compartment in both lymphocytic and myelogenic lineages. To a lesser extent, it can also be expressed by other cell types of the non-hematopoietic compartment, such as skin, keratinocyte, and osteoblasts (47). NLRP3 consists of a carboxy-terminal LRR domain, a central NOD domain, and an amino-terminal PYD, mainly interacting with apoptosis-associated speck-like protein containing a CARD (ASC) (8) (Figure 1). NLRP3 participates in inflammasome formation through the recruitment of ASC, subsequent activation of caspase-1, and secretion of IL-1β and IL-18. Activation of inflammasomes is thought to involve two steps. The first, or priming signal, involves the transcription of pro-IL-1β, pro-IL-18, and pro-caspase-1, as well as expression of NLRP3 itself. The second step, or activating signal, involves mechanisms that lead to inflammasome assembly in response to microbial or danger signals, and in turn drives cleavage and release of biological active IL-1β and IL-18. Hence, mice lacking caspase-1 are defective in the maturation and secretion of IL-1β and IL-18 (48–50). NLRP3 is able to recognize a variety of exogenous and endogenous stimuli including lipopolysaccharide (LPS), MDP, bacterial, and viral RNA (51–53) as well as the imidazoquinoline antiviral compounds R837 and R848 (54) (Table 1). *In vitro*, addition of ATP to macrophages that are pre-exposed to TLRs ligands such as LPS can significantly increase caspase-1 activation and secretion of IL-1β. Importantly, ATP stimulates the P2X_7_ receptor, which results in the opening of a pore mediated by hemichannel protein pannexin-1. A consequence of this process is that, following stimulation with ATP bacterial products are able to enter the cytosol through the pore and to subsequently activate NLRP3 and caspase-1. Thus, NLRP3 recognition of PAMPs appears to be linked to pannexin-1-dependent pore formation (55, 56).

Polymorphisms within the NLRP3 gene have been associated with several disorders, including celiac disease, psoriasis, type 1 diabetes, and increased susceptibility to HIV-1 (57–59). SNPs in the *NLRP3* regions were originally reported to be associated with CD, however, other groups have not detected this association (60). However, SNPs affecting receptors downstream from NLRP3, such as IL-18R1, IL-1RL1, IL-1RL2, and IL-1R2, have been associated with increased IBD susceptibility (56). In addition, polymorphisms within the gene encoding the inflammasome effector cytokine IL-18 correlate with increased susceptibility to CD (61). In this context, understanding the mechanisms by which inflammasome effector cytokines IL-1β and IL-18 modulate gut homeostasis is of particular importance. In fact, intestinal mucosa cells located in affected areas of the gut from patients with CD display elevated expression of IL-18 (62, 63). Notably, IL-18 derived from intestinal epithelial cells during the initial phase of intestinal inflammation has been demonstrated to play a protective role in facilitating tissue repair and supporting homeostatic mechanisms. In fact, both IL-18 and IL-18R deficient mice are more susceptible to acute DSS-induced colitis than wild-type control mice. Interestingly, epithelial-derived IL-18 is also critical for protection against DSS colitis conferred by NLR-mediated signaling, as shown in studies using NLRP3 deficient mice (64). In contrast, during the chronic phase of intestinal inflammation, production of IL-18 shifts from epithelial cells to lamina propria monocytes. This cellular redistribution may be responsible for the change to a pro-inflammatory role that IL-18 plays during chronic inflammatory responses within the gut mucosa. Similar to IL-18, intestinal mucosal IL-1β levels are also elevated in IBD (65). IL-1β plays a pro-inflammatory role, as its neutralization by either endogenous or exogenous administration of IL-1R antagonist results in significant amelioration of colitis (66–68). However, administration of recombinant IL-1β also has a beneficial effect, suggesting that IL-1β is fundamental for mucosal protection and maintenance of homeostasis (69). These protective effects were only achieved with administration of a low dose of IL-1β, and only when given 24 h before induction of colitis. Neutralization of IL-1β activity during the acute phase of disease was associated with exacerbated severity of inflammation and delayed recovery from injury (70). Thus, it appears that homeostasis of the intestinal mucosa is highly sensitive to the levels of expression of the inflammasome effector cytokines IL-1β and IL-18, and dysregulated expression that results in either overproduction or underproduction of these cytokines proteins may severely affect the susceptibility of the gastrointestinal tract to chronic inflammation.

Recent studies examining the molecular mechanisms by which NLRP3 and caspase-1 control integrity of the intestinal epithelium during experimental colitis point to a critical role of NLRP3 in gut mucosal immune homeostasis (71). Specifically, NLRP3 deficient mice were significantly more susceptible to DSS colitis compared to wild-type mice (64, 71, 72), and deficiency of the inflammasome proteins ASC and caspase-1 caused greater colitis-associated mortality and more severe inflammation during both the acute and chronic phases of colitis (64, 71, 73). One possible explanation is that following chemically induced insult on the intestinal epithelium, NLRP3 inflammasomes may trigger repair mechanisms characterized by increased division of stem cells at the base of crypts to replace damaged enterocytes (74). In addition, the absence of NLPRP3 led to defective production of the downstream effectors cytokines IL-18 and IL-1β, resulting in increased permeability of the gut epithelium. Compromised epithelial barrier function in both NLRP3 and caspase-1 deficient mice allows bacteria to invade the intestinal lamina propria and mucosa, which accelerates inflammatory responses and leads to chronic intestinal inflammation (75).

Together, these studies indicate that NLRP3 plays a central role in regulating the integrity of the intestinal mucosal barrier under homeostatic conditions, and in shaping innate immune responses during experimental colitis. In addition, NLRP3 effector cytokines IL-1β and IL-18 play dual roles such that their dysregulated expression may have both protective or pro-inflammatory effects, depending on the distinct phase of disease.

### NLRP6

NLRP6 is high expressed in the duodenum, ileum, and colon, primarily within the hematopoietic compartment by macrophages, dendritic cells, lymphocytes, and granulocytes (74, 76, 77). NLRP6 signaling is important in both radioresistant (stromal) and radiosensitive (hematopoietic) cells during bacterial infection with *Salmonella typhimurium, Listeria monocytogenes*, and *Escherichia coli* (78). The structure of NLRP6 is less well characterized than other members of the NLR family and consists of an N-terminal PYRIN domain, a central nucleotide-binding domain (NBD), and a C-terminal LRR (Figure 1). NLRP6 co-localizes with the ASC, and this is directly dependent on the presence of the PYRIN domain (79, 80). Co-expression of NLRP6 and ASC results in cooperative induction of caspase-1 and increased production of IL-1β, suggesting that NLRP6 participates in inflammasome signaling (79). Reduced expression of IL-18 was observed in NLRP6 deficient mice, as well as increased susceptibility to chemically induced colitis (81). However, direct evidence that NLRP6 activates *in vivo* inflammasomes under physiological conditions has not been fully elucidated. In addition, NLRP6 is positioned upstream from both NF-κB and MAPK signaling pathways, such that NLRP6 deficient mice enhance both pathways upon stimulation with TLR2 and TLR4 ligands *in vitro*. NLRP6 deficient cells that are infected with bacteria produce increased levels of TNF-α and IL-6, suggesting that NLRP6 negatively regulates innate immunity and host defense against both Gram-positive and Gram-negative bacteria (82).

As already mentioned, NLRP6 is highly expressed in the intestine and may play a central role in maintaining homeostasis within the intestinal mucosa. Moreover, NLRP6 is protective against the development of significant damage and inflammation within the colon during chemically induced DSS colitis. Interestingly, microbiome profiling, as analyzed by 16S RNA sequence analysis, revealed that in mice deficient in NLRP6, ASC, caspase-1, or IL-18, gut microbial ecology is altered with a predominant role of Prevotellaceae and Tm7 as key representative members of this microbiota-associated phenotype; these strains have also been found to be increased in IBD patients (81). This altered microbiota was associated with a colitogenic phenotype, which was transmissible to wild-type mice housed in the same cages. Notably, the use of antibiotics ameliorated the DSS-induced colitis, further supporting the colitogenic activity of Prevotellaceae. NLRP3 deficient mice also have decreased production of IL-18, suggesting that the altered microbiome in these mice is directly related to IL-18 levels. In fact, IL-18 deficient mice were also able to transmit colitogenic flora to wild-type mice.

Of note, changes in the microbiome not only play a critical role in determining susceptibility to colitis, but also to inflammation-associated tumorigenesis (83). In fact, in the AOM–DSS model of inflammation-induced tumorigenesis, NLRP6 deficient mice developed significantly more tumors and hyperplasia compared to wild-type mice, and this effect was associated with elevated levels of CCL5. Interestingly, co-housing experiments using NLRP3 and CCL5 deficient mice did not result in increased colitis or tumors in CCL5 deficient mice, suggesting that the colitogenic and tumorigenic microbiota may be mediated by the presence of CCL5 (84). Altogether, these results strongly suggest that a dysregulated NLRP6 inflammasome pathway may be a predisposing factor to chronic intestinal inflammation and inflammation-associated tumorigenesis.

### NLRP12

NLP12, previously known as monarch-1 and PYPAF7, was one of the first NLR proteins to be described and was originally associated with inflammasome formation (79, 85). NLRP12 co-localizes with ASC, dependent on the presence of the PYRIN domain, similar to NLRP6 (80). *In vitro* studies originally demonstrated that transient transfection of NLRP12 and ASC induces inflammasome formation, as well as transcription of an NF-κB reporter construct, suggesting that NLRP12 was an inflammasome forming NLR, and promoted caspase-1 activation and production of IL-1β, in addition to being a positive regulator of NF-κB signaling (72). Evidence for the involvement of NLRP12 in induction of inflammasome formation are mostly based on *in vitro* studies; *ex vivo* studies have evaluated NLRP12 inflammasome formation using NLRP3 deficient mice, and have shown that this NLR actually is not able to regulate formation of either IL-1β and IL-18 (73–75, 86–90).

Notably, several studies have demonstrated that NLRP12 down-regulates NF-κB responses to TLR agonists (75, 77, 78, 86, 88, 89, 91–93). Specifically, previous *in vitro* studies showed that NLRP12 negatively regulated non-canonical NF-κB pathway activation by directly associating with TRAF3 and NF-κB inducing kinase (NIK) (75, 94). This interaction leads to proteasome-mediated degradation of NIK and subsequent attenuation of p100 cleavage to p52 (95). Also, NLRP12 has been shown to attenuate canonical NF-κB signaling through inhibition of IRAK-1 (75, 91, 92) and to down-regulate the MAPK cascade by attenuation of ERK signaling activation (75, 89).

As observed for NLRP6, NLRP12 plays a central role in protecting against chemically induced colitis and inflammation-associated tumorigenesis (75, 89). In fact, NLRP12 deficient were more susceptible to DSS-induced colitis and AOM-DSS-induced colon cancer. However, NLRP12 is not involved in regulation of the microbiome, as NLRP12 deficient mice are unable to transmit colitogenic flora to wild-type mice after co-housing, in contrast to what it has been observed for NLRP6 (81). The increased inflammatory responses observed in NLRP12 deficient mice during DSS-induced colitis are correlated with increased canonical and non-canonical NF-κB activation signaling. In addition, tumors isolated from these mice showed significantly higher non-canonical activation of NF-κB and increased expression of inflammation and cancer-related markers (75, 89). Using bone marrow chimera mice, Allen et al. suggested that both the hematopoietic and non-hematopoietic compartments contribute to early disease manifestations during DSS-induced colitis, however, the non-hematopoietic compartment is important for limiting the number of tumors formation (75).

In summary, these studies provide evidence of an anti-inflammatory and anti-tumorigenic role for NLRP12 at the intestinal level. These effects have been mostly associated with down-regulation of NF-κB and ERK signaling activation. Therefore, further understanding of the role of NLRP12 may help identify new therapeutic approach aimed at stimulating this factor in order to potentially control intestinal inflammation and inflammation-associated tumorigenesis.

### NLRC4

NLRC4, also known as IPAF, is mostly expressed in myeloid cells. NLRC4 contains an N-terminal CARD domain, a central NACHT domain, and a C-terminal LRRs (Figure 1). The N-terminal CARD allows a direct interaction with caspase-1, independently of ASC. NLRC4 can activate the caspase-1 inflammasome upon cytosolic detection of bacterial flagellin, but also components of the type III secretion system (T3SSs) in associations with NAIP proteins (96) (Table 1). In addition to cytokine production, activation of caspase-1 through a NLRC4 dependent pathway has been associated with subsequent death of cells, termed pyroptosis, which can take place independent of ASC (97, 98). Therefore, although there are structural similarities with other NLR proteins, activation of NLRC4 may have an opposite function in determining the activity of caspase-1, adding more specificity to the function of the innate immune system in response to bacteria.

NLRC4 plays a central role in host defense following infection with several pathogens, such as *Legionella pneumophila, Candida albicans, S. typhimurium, Burkholderia pseudomallei*, and *Pseudomonas aeruginosa* (99–102). In fact, all of these pathogens lead to caspase-1 activation, release of IL-1β, and rapid cell death. For example, NLRC4 deficient macrophages infected with *S. typhimurium* showed defective activation of caspase-1 and secreted IL-1β and IL-18 (103). In addition, *S. typhimurium*-induced macrophage death was also retarded in NLRC4 deficient macrophages. In another study, Franchi et al. reported that NLRC4 deficient mice were highly susceptible to orogastric *Salmonella* infections (104). In this study, the authors found that NLRC4 deficient mice with the Balb/c genetic background, but not the C57BL/6 background, were highly susceptible to orogastric infection with *Salmonella*, suggesting a central role for the NLRPC4 in host defense against enteric *Salmonella* through the production of IL-1β by resident intestinal macrophages (104).

Most of these studies have concentrated on the function of NLRC4 within the hematopoietic compartment, primarily in macrophages. However in a recent study using NLRC4 deficient mice, it has been shown the NLRC4 plays a protective role against infection with *Citrobacter rodentium* that is dependent on its expression in non-hematopoietic cells. *Citrobacter rodentium* is an extracellular pathogen that adheres to the intestinal epithelium of the large intestine causing hyperplasia and inflammation (105). Specifically, bone marrow chimera experiments revealed that the protective effect of NLRC4 is dependent on its elevated expression in epithelial crypts, but not in intestinal stromal cells (105). Another study supports an intrinsic epithelial cell effect leading to enhanced tumorigenesis in the absence of NLRC4 (84). In fact, this study showed that NLRC4 mice have a higher frequency of tumors in the AOM–DSS model compared to wild-type mice. Therefore, further studies of these mechanisms need to be conducted to enhance our knowledge on the role of NLRC4 in epithelial repair to an acute inflammatory insult.

## Class II Transactivator

Class II transactivator is a member of the NLR/CATERPILLER family of proteins consisting of a series of regulatory domains that include an activation domain (AD), an acetyl-transferase (AT) domain, a proline/serine/threonine (PST) domain, a GBD, and finally, the canonical LRR domain common to all NLR proteins (106) (Figure 1). Upon stimulation with IFN-γ, STAT-1 triggers CIITA expression though IFN-γ activation sequence elements present in the CIITA promoters. Distinct from others NLRs, the function of CIITA lies in transcriptional regulation of the MHC (107–110) (Table 1). In fact, it induces transcription of MHC class II genes and enhances constitutive MHC class I gene expression. CIITA is mainly expressed in cells that express MHC class II molecules, such as lymphocytes, macrophages, and dendritic cells. In addition, CIITA has been demonstrated to negatively regulate innate immune responses by regulation of NOD2 (107).

Although CIITA is well-known as a key regulator for the expression of MHC class II molecules, it also plays an additional role in T-helper cell differentiation and activation-induced cell death (111). Expression of CIITA in T cells enhances Th2-type immunity, as shown by *in vivo* studies where transgenic mice expressing CIITA (CIITA-tg mice) had increased susceptibility to oxazolone-induced colitis, an experimental model of Th2 mediated intestinal inflammation. This study found that CIITA expression in CD4^+^ T cells tends to bias CD4^+^ T cells toward Th2-type immune responses, inhibiting Th1 differentiation (111).

A more recent study demonstrated that silencing one of the four CIITA promoters, the CIITApIV, which is inducible by IFN-γ, causes the loss of MHC class II expression on tumor cells. A study using gastric and colorectal tumor cell lines demonstrated that a subset of these tumor cells do not induce CIITA expression following IFN-γ stimulation (112). Interestingly, it has been reported that an association exists between CIITA methylation and DR17 and DQ2 HLA alleles in patients with UC and colon-associated cancer (CAC) (113).

## Conclusion

During the last decade accumulating evidence led to the novel hypothesis that defective innate immune responses at the intestinal level may be the primary contributor to IBD. The most compelling support for this concept derives from the clear genetic associations between CD and carriage of polymorphisms within the *NOD2* gene, further underscoring the importance of these PRRs in IBD. In this context, several studies discussed in this review described how multiple members of the NLRs protein family regulate intestinal inflammation. Activation of these pathways is mediated by bacterial components in order to generate acute inflammatory responses that lead to bacterial clearance. Thus, down-regulation of these signaling pathways during the early phases of disease may predispose to chronic intestinal inflammation. In contrast, inappropriate activation of these signaling pathways during chronic phases of intestinal inflammation may result in continuous production of pro-inflammatory mediators that contribute to maintaining chronic intestinal inflammation. Therefore, a better understanding of the dichotomous nature of NLRs during acute and chronic phases of intestinal inflammation may provide new insights into therapeutic strategies against IBD.

## Conflict of Interest Statement

The authors declare that the research was conducted in the absence of any commercial or financial relationships that could be construed as a potential conflict of interest.
